# Modeling Saudi stock index returns and volatility: a dual approach using GARCH and neural networks

**DOI:** 10.3389/frai.2026.1714822

**Published:** 2026-03-04

**Authors:** Sukainah AL-Besher, Dania AL-Najjar

**Affiliations:** Department of Finance, School of Business, King Faisal University, Al-Ahsa, Saudi Arabia

**Keywords:** GARCH, LSTM, return series, volatility, forecast, stock index, Saudi stock market

## Abstract

The financial markets are the drivers of economic growth as they organize savings, bring in foreign investment, and they efficiently allocate resources. The Tadawul is the largest stock market in the GCC, which is highly impacted by prices of oil and gold. Correct forecasting of market returns and volatility is thus a key to investors and policymakers. The research analysis is performed on the Tadawul All Share Index (TASI) between January 1, 2000, and December 31, 2022, with the help of traditional GARCH-family models (GARCH, EGARCH, GJR-GARCH, and MGARCH) and a Long Short-Term Memory (LSTM) neural network. Explanatory variables include oil and gold returns, MSE, MAE, RMSE, R 2, and Diebold-Maasoony test are used to assess predictive performance. The results show that the LSTM model is the most effective model that captures nonlinear volatility patterns, whereas GARCH models, especially the GJR-GARCH model with GED distribution offers better returns projections. The findings also confirm that oil and gold returns have a significant influence on the performance of TASI, which proves their role in the oil-dependent economy. Altogether, the evidence demonstrates the synergistic advantages of both econometric and machine learning methods and provides useful implications of risk management and investment decision-making as well as policy guidance.

## 1 Introduction

The significance of financial markets to the economic growth in the country can be explained by the fact that the financial markets are the ones that welcome national savings and foreign capital. They also provide an effective distribution of resources. Quantitative models, which are more sophisticated, have been borrowed by financial markets. The main aspects of risk management and the most appropriate decision-making in investments are market returns and the dynamics of market volatiliti (Kamakhli and Katlo, 2020; Al-Tijani et al., 2020). KSA is located in one of the Gulf Cooperation Council (GCC) single markets. This market is characterized by huge and expanding economies replacing the world market through production of oil. The industry is mainly reliant on the energy prices. Hence, the value of proper predictions of the stock market to policy makers and investors is pronounced (Youssef et al., 2021; Bekhet et al., 2017). The Saudi Stock Exchange (Tadawul) is the largest in the GCC region and the MENA region due to good economic fundamentals, an established banking system, and structural reforms that have been enabled by Vision 2030. On the contrary, Tadawul is highly dynamic in terms of the impact of the internal and external factors, including the oil price fluctuations and the financial situation of the globe. It is a tough market where the performance can be predicted. It requires more advanced instruments of analysis (Kamakhli and Katlo, 2020). Most of the studies have evaluated the volatility of Tadawul using univariate techniques. Such research neglected the important externalities, including oil and gold prices (Alamro et al., 2019; Alhagyan and Alduais, 2020). Although they had applied the traditional econometric models, such as the GARCH, in the analysis, the most significant issue with the models is the fact that they had not included the significant extraneous variables, such as the oil and gold prices. Besides this, the weakness of the study is that the classical econometric and modern machine-learning strategies are not explored. These methods may characterize nonlinear relationships and many-sided dependence of time. The present paper will fill these gaps. By using GARCHs and Long Short-Term Memory (LSTM) neural networks, the paper will determine the returns and volatility of the Saudi Stock Exchange (Tadawul) All Share Index (TASI) over the time period of January 1, 2000, to December 31, 2022. The reason why LSTM will be selected is that it has already been demonstrated that they can demonstrate the dependence of time change of a long-term nonlinear dynamics of financial time series. They particularly have a fit in the volatile and oil market. Later architectures, such as Transformers, have also been found to have financial forecasting potential. Nevertheless, LSTM is highly applicable when making financial predictions in long series and financial markets where comparatively smaller volumes of information can be accessible in high frequency. The oil and gold prices are used in the model as the explanatory variables in order to establish the performance of the model, as well as to examine the economic impact of manipulating the prices of the energy to give the policy makers and investors the theoretical and practical information. With the recent developments in the market dynamics in the post-2022 year, the changes in the oil prices, the tendencies in the post-pandemic rebound, etc., it has become even more apparent that attractive forecasting models could be applied to the Saudi stock market. There are nine crucial sections in the paper. These include Introduction, Literature Review, Data, Methodology, Results and Discussion, Conclusions, Practical and Theoretical Implications, Future Work, and the Limitations.

## 2 Literature review

In this section, previous studies are summarized into five subsections: Price Forecasting and Return and Volatility Modeling, Econometric Models, and Engineered Learning and Deep Learning, Behavior of the Market Oil and Gold, GCC Markets and Saudi Arabia Evidence, and Knowledge Gaps and Motivation.

### 2.1 Price forecasting and return and volatility modeling

The stock market prognostication literature comprises two general strands: the price-level forecasting and the return-volatility modelling strands. The aim of price forecasting is to predict the future price trend to short-term trade and verify their accuracy. Neural networks (NN), support vectors machines (SVM), and ensemble models are used in most of those works to maximize the accuracy of point forecast (Huang et al., 2023; Singh and Jain, 2022; Khoa et al., 2023). On a case in point, LSTM and other recurrent designs are seen to be rather effective at nonlinear temporal dynamics of short-term horizons. Nevertheless, risk measures and volatility characteristics are seldom reported by them. However, a study of risk dynamics, market efficiency, and asset allocation cannot be done based only on price forecasting because it usually assumes no attention to return distributions, as well as to conditional volatility. These are the main aspects of the contemporary asset pricing and risk management. Moreover, there are also numerous studies which employ point prediction measures (MAE, RMSE). They make this without evaluating statistical adequacy or the economic significance of predictions, making them limited in risk-sensitive contexts. Comparatively, the distributional properties of returns and conditional heteroskedasticity are directly estimated using return volatility models. The conclusion can be made on persistence, clustering, and shock transmission using them. These characteristics make the difference in efficient portfolio and policy making. The strand takes the following conventional econometric specifications and hybrid machine learning as their assumptions.

### 2.2 Econometric models and engineered learning and deep learning

Econometric models prevail in volatility modeling, in particular, in the GARCH family, due to their theoretical foundation and capacity to interpret. Different markets exhibit systematic clustering and leverage effects, which are systematically represented by symmetric (GARCH) and asymmetric (EGARCH, TGARCH) variants (Naseem et al., 2018; Nguyen and Nguyen, 2019; Yousef, 2020). Such models can also be tested with regard to the stability of processes and persistence of shocks. This cannot be clearly seen using several black-box techniques. Nonlinear dependencies and long-run dynamics, however, are also a problem in GARCH models. This is particularly with non-stationary or volatile conditions, such as in the case of a crisis. This has limited the studies to machine learning (ML) and deep learning (DL). Various forms of econometric structure hybrid models with neural work are typically better than isolated GARCH in regard to capturing complex patterns. To explain, hybrid GARCHNN models have a greater volatility prediction rate. It can be explained by the advantages of nonlinear pattern recognition as well as statistical framework (Liao et al., 2020; Koo and Kim, 2022). This tendency is continued subsequently in later research (2024–2025) through the assistance of transformer-based architectures and attention mechanisms, as well as state-space models. Transformer models are self-attention models, based on long-term dependencies instead of RNNs, e.g., LSTM. They give superior predictions on the currency and equity volatilities issues (Rahman et al., 2025). Transformers systems can also be improved with regards to predictive performance due to Time2Vec-based transformers systems. They are trained on a set of various periodic patterns of financial time series (Srivastava, 2025). Multi-transformer designs are also characterized by the capacity to perform properly in various market regimes (Mishra et al., 2024). Hybrid FEDformer models are used to increase anomaly detection and risk prediction of processes in long history (Fan et al., 2025). These tendencies remind us of the ever-changing volatility forecasting beyond the more classical econometric methods. They, however, usually require huge amounts of data. They can also fail to be interpretable as they would be wished.

### 2.3 Behavior of the market oil and gold

The behavior of stock market is also superb to other external macro-drivers such as the price of crude oil and gold. This is more so in resource-based economies. It is frequently documented in many works that the spillover effects of oil prices on the market returns and volatility are significant (Salisu et al., 2022; Liu et al., 2023). The impacts of gold on the regimes and horizons are also not purely positive (Yuan, 2023; Patalay, 2022). Despite this evidence, many volatility models do not contain such drivers. Instead, they merely make them a linear part. This may lead to unsuitable volatility relations and biased perspective. Even those papers which study the influence of oil and gold tend to do the latter outside of advanced nonlinear models. This is another loophole that is closed in the present study.

### 2.4 GCC markets and Saudi Arabia evidence

Specific studies of the Saudi Stock Exchange (TASI) are not very sophisticated. ARIMA, ANN, RNN, or hybrid versions of ML are most frequently used in the studies to predict prices. Instead, they are doing so in contrast to generating joint returns-volatility models (Alotaibi et al., 2018; Jarrah and Salim, 2019; BinMakhashen et al., 2022). The models can be useful in predictive accuracy on short-term horizons. They, however, do not give many details about conditional risk structure. The GARCH volatility model used in TASI also has limitations with regard to length of the sample or the model characteristics. It gives inconclusive results regarding leverage influences and crisis responses (Shaik and Syed, 2019; Wasiuzzaman, 2022). The asymmetric impacts and the effect of pandemics are occasionally reported in studies across the GCC. Nonetheless, they rarely use ML models and project oil and gold in advanced volatility models (Abou Elseoud and Haji, 2023; Elhassan, 2021).

### 2.5 Knowledge gaps and motivation

As this review shows, there are certain significant gaps in the literature. The former is the lack of clarity of concept and methodology between price projections and the return-volatility modeling. The reports are extremely few that provide a robust blend of econometric and deep learning models. This is in the general backdrop of a forecasting model that includes dynamic of both the returns and the volatility. Majority of the models take them separately. Alternatively, they fail to integrate them into complex nonlinear forecasting models. This is regardless of their structural importance, particularly oil and gold. The current study provides answers to these gaps by estimating returns and volatility of TASI concurrently with GARCH-family models and LSTM. It compares them to the performance of transformer-based and hybrid models. It also explicitly includes the crude oil and gold returns as a side variable over a long historical time.

## 3 Data

This section demonstrates the information, statistical features, and analysis. It is constructed to address the requirements of GARCH-based and LSTM-based models, all the diagnostic checks, and data pre-processing.

### 3.1 Data description

In financial studies, prices are not preferred because of inappropriate statistical properties (Costa, 2017): The returns are not normally distributed, skewed, and tailored. The returns have an insignificant serial correlation. There is positive dependence of absolute returns, which means that the volatility is concentrated.

Variables: TASI daily log-returns. Gold and crude oil (WTI) between January 1, 2000, and December 31, 2022. These were the findings of the Investment site (www.investment.com). Cleaned and standardized data were then created: no-trading days were eliminated. The results of cleaning and normalization of the data were as follows: Non-trading days were eliminated. The linear interpolation was used to fill in the missing values. Extreme levels were trimmed down to the outliers. Data leakage Prevention: Min-Max method of normalization of training data and test data using training data parameters.

#### 3.1.1 Return definition

rt=ln(Pt)−ln(Pt−1)

where P_t_ is the closing price on day t.

### 3.2 Diagnostics and descriptive statistics

Table 1 shows that Gold and TASI returns are the same, with each amounting to 0.000540. They are much higher than the Crude Oil return, which reaches −0.000099. According to the maximum returns of the three variables, the analysis shows that crude oil returns have the highest value of 0.37662 compared with gold returns, with the lowest value of 0.11345.

**Table 1:** Variables.

Variable	Type	Period	Source
TASI	Dependent	Jan 1, 2000 – Dec 31, 2022	www.investment.com
Gold	Independent		
Crude oil (WTI)			

Source: Prepared by the researcher.

**Figure 1:**
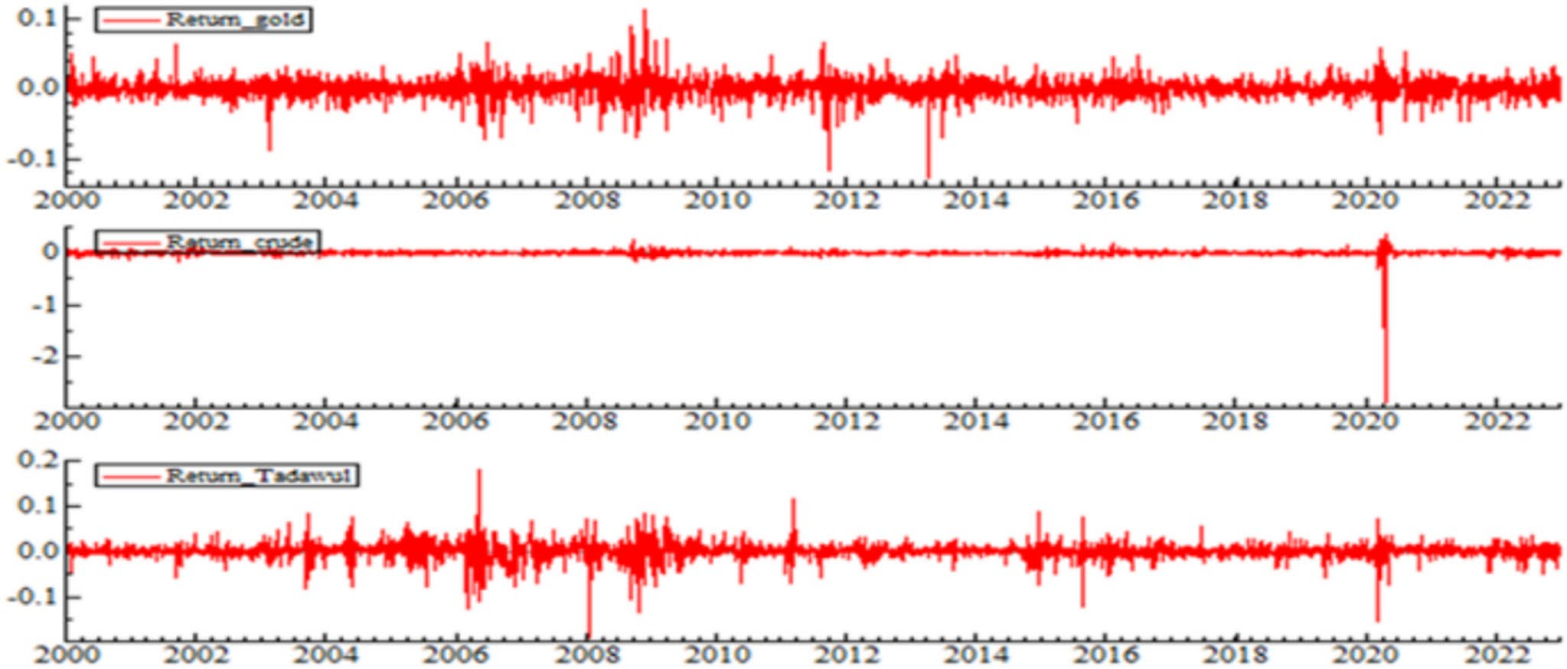
TASI, gold, and crude oil daily log-returns.

Each variable exhibits a large gap between max and min values, which indicates high price volatility. The skewness values of the variables in this study are negative, and the kurtosis values are higher than 9. Therefore, the distributions of these variables are leptokurtic and fat-tailed. Negative skewness coefficients are obtained for the three variables, which suggests that bad news affects the returns with higher rates than good news. In other words, the behavior of financial market investors depends heavily on any previous event or shock. The kurtosis coefficients are greater than 3 for all variables, which means that these variables exhibit excessive and high kurtosis. The median calculations rank the variables as follows: crude oil returns, TASI, and gold returns. However, the volatility, as measured by standard deviations, shows that crude oil has the highest volatility, followed by TASI returns, and gold returns come last. The volatility in the variables or the fluctuations in the returns can be observed in Figure 1.

The Jarque-Bera test is statistically significant at a 5% level, which indicates that returns are not normally distributed. Thus, all the previous statistical results support advancing the development of GARCH models for TASI returns. Accordingly, the distributions of the three variables are leptokurtic and asymmetric, with excessive fluctuations.

The stationarity of each variable is tested using one of the unit root tests to forecast the volatility and returns of TASI using GARCH and LSTM models. These tests help determine the appropriate method that can test or make the time series stationary. The researchers conduct one unit root test, the developed Dickey-Fuller (DF) test. This methodology aligns with those of Tamilselvan and Vali (2016), Fwaga et al. (2017), Naseem et al. (2018), Mohsin et al. (2019), Marobhe and Pastory (2019), Sekati et al. (2020), Endri et al. (2021), Alzyadat et al. (2021), and Ampountolas (2021).

The augmented DF (ADF) test is shown in Table 2. The results are applied to the three (i.e., 1, 5, and 10%). The findings show that the data of all variables are stationary, with a probability of less than 5%, which confirms the absence of autocorrelation.

**Table 2:** Descriptive statistical analysis.

Variables	Gold	Crude oil WTI	Tadawul all share	Null hypothesis
Observations	4,116	4,116	4,116	
Mean	0.00054	-9.90E-05	0.00054	
Standard deviation	0.013207	0.058199	0.016839	
Skewness	−0.23486	−32.255	−1.0337	
Excess Kurtosis	9.444	1537.9	16.687	
Minimum	−0.12683	−2.8938	−0.18916	
Maximum	0.11345	0.37662	0.18046	
Median	0.000365	0.001316	0.001157	
Normality test: (Jarque-Bera test)	3781.8***	1951***	5506.5***	Normal distribution
ADF	−68.02***	−50.88***	−62.15***	Unit root = 1
Arch-LM	23.5***	144.4***	122.9***	No ARCH effect
Significant values: 0.10 *, 0.05**,0.01***				

Source: Prepared by the researcher based on the results of statistical analysis.

After the stationarity of the time series data is verified, the ARCH effect is tested for each variable. This step is important to test the homogeneity of error variances and thus verify the possibility of building the GARCH model. As shown in Table 2, the ARCH-LM test results (23.5**, 144.4**, 122.9**) indicate ARCH effects, suggesting that there is autoregressive conditional heteroskedasticity in the returns of these assets. Thus, the possibility of building GARCH models exists.

The data analyzed dataset contains 4,116 observations for all variables: gold returns, crude oil returns, and TASI returns. This sizable dataset allows for a thorough examination of the relationships among all variables over the period covered by the 4,116 observations.

### 3.3 Diagnostics of long-memory and exogeneity

Hurst Exponent Values that are above 0.5 confirm long-memory behavior of any series.

Granger Causality Test (oil and gold returns, TASI returns): Both of the two variables can be an exogenous variables.

### 3.4 Visualization

Figure 1 in the visual examination of the Gold, crude oil, and TASI returns indicates that the returns of the three fluctuate daily. Such dispersion indicates that returns are clustered together and gives a long memory argument. According to this outcome, huge (minor) changes are likely to be pre-followed by big (minor) changes.

**Figure 2:**
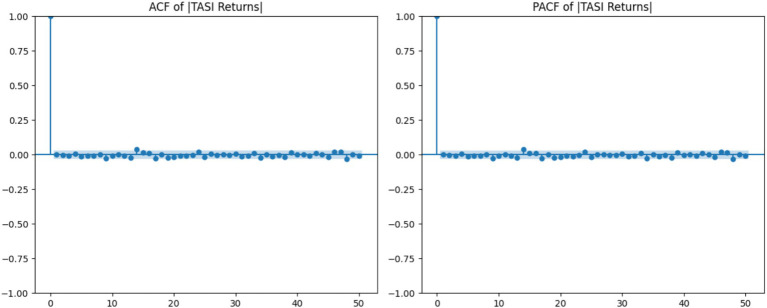
ACF and PACF of absolute returns.

According to Figure 2, it can be seen that the autocorrelation and partial autocorrelation (ACF/PACF) plots indicate that there is persistence in the absolute returns, confirming the volatility clustering.

### 3.5 Key modeling implications

The use of log-returns is done to provide statistical robustness. Time series are stationary (ADF test). Effects of ARCH justify the GARCH. Time-dependent model diagnostics are facilitated by long-memory diagnostics. The oil and gold returns are exogenous, and that is reasonable as explanatory variables. The normalization is applied appropriately to prevent data leakage in the ML models. Overall, the information can be used in the high-level volatility models, econometric (GARCH), and deep-learning-based (LSTM, transformer-based).

## 4 Methodology

In this section, provide the description of the econometric and deep learning models that are applied to model and predict TASI returns and volatility. The proposed model will rely on both exogenous variables and LSTM neural networks to utilize both the linear dynamics of the volatility and nonlinear relationships with the help of GARCH-family-based models. The methodology is constructed in a manner that it provides the work with theoretical conformity, empirical power, and full repeatability.

### 4.1 GARCH models

Bollerslev (1986) further notes that Generalization Autoregressive Conditional Heteroskedasticity (GARCH) is a popular model that is used to model and predict time-dependent volatility in financial time. GARCH models allow conditional variance to be dependent on historical squared innovations, and conditional variances lagged, and therefore are quite successful in capturing volatility cluster that is remarkably prevalent in the asset markets (Tamilselvan and Vali, 2016; Fwaga et al., 2017; Naseem et al., 2018; Mohsin et al., 2019; Marobhe and Pastory, 2019; Sekati et al., 2020; Endri et al., 2021). Empirical studies confirm that GARCH models are more useful compared to the traditional ARCH model and other linear volatility models in predicting returns (Costa, 2017).

The conditional variance equation is specified as:


$$\sigma_{t}^{2}=\omega +\sum_{j=1}^{q}\alpha_{j}\varepsilon_{t-j}^{2}+\sum_{i=1}^{p}\beta_{i}\sigma_{t-i}^{2},$$

where:

-
$\sigma_{t}^{2}$ is the conditional variance of returns.
-
ω>0 is a constant.
-
$\alpha_{j}\geq 0$ capture the short-run impact of shocks (ARCH effects).
-
$\beta_{i}\geq 0$ measure volatility persistence (GARCH effects).
-
p and q denote the orders of the GARCH and ARCH terms, respectively.

### 4.2 GARCH family extensions

To consider the asymmetry and leverage, some of the GARCH variants are considered:

#### 4.2.1 GARCH-in-MEAN (GARCH-M)

$$r_{t}=\mu +\lambda \sigma_{t}^{2}+\sum_{i=1}^{k}\phi_{i}r_{t-i}+\varepsilon_{t},$$

where λ reflects the risk–return trade-off.

#### 4.2.2 Exponential GARCH (EGARCH)

$$\ln(\sigma_{t}^{2})=\omega +\sum_{j=1}^{p}\beta_{j}\ln(\sigma_{t-j}^{2})+\sum_{i=1}^{q}\alpha_{i}(\frac{|\varepsilon_{t-i}|}{\sigma_{t-i}}\sqrt{\frac{2}{\pi }})+\sum_{i=1}^{q}\gamma_{i}\frac{\varepsilon_{t-i}}{\sigma_{t-i}},$$

where $\gamma_{i}$ captures asymmetric responses to positive and negative shocks.

#### 4.2.3 TGARCH/GJR-GARCH

$$\sigma_{t}^{2}=\omega +\sum_{i=1}^{q}(\alpha_{i}+\gamma_{i}I_{\left\{\varepsilon_{t-i}<0\right\}})\varepsilon_{t-i}^{2}+\sum_{j=1}^{p}\beta_{j}\sigma_{t-j}^{2},$$

where I. is an indicator function equal to 1 for negative shocks and 0 otherwise.

The previously mentioned models are commonly used in financial econometrics and have been proven to be effective in modeling and forecasting financial volatility. (Tamilselvan and Vali, 2016; Fwaga et al., 2017; Naseem et al., 2018; Mohsin et al., 2019; Marobhe and Pastory, 2019; Sekati et al., 2020; Alzyadat et al., 2021; Ampountolas, 2021).

### 4.3 GARCH models with exogenous variables by conditional mean equation (ARX specification)

The ARX process is used to model the conditional mean of the returns of TASI, such that the exogenous variables of the model are oil and gold returns.


$$r_{t}=\mu +\sum_{i=1}^{k}\phi_{i}r_{t-i}+\delta_{1}r_{t}^{\text{oil}}+\delta_{2}r_{t}^{\text{gold}}+\varepsilon_{t},$$

where:

-
$r_{t}$ denotes the return of TASI at time t .
-
μ is a constant term.
-
$\phi_{i}$ are autoregressive coefficients.
-
$r_{t}^{\text{oil}}$ and $r_{t}^{\text{gold}}$ represent crude oil and gold returns, respectively.
-
$\delta_{1}$ and $\delta_{2}$ measure the impact of oil and gold returns.
-
$\varepsilon_{t}$ is the error term, assumed to satisfy $\varepsilon_{t}=z_{t}\sigma_{t}$ , with $z_{t}$ following a standardized distribution.

This assertion gives a straight relationship between returns generation process and the volatility dynamics as modeled by GARCH dynamic (Tables 3, 4).

**Table 3:** Hurst exponent estimates.

Variable	Hurst exponent (H)
TASI	0.64
Gold	0.61
Crude Oil	0.69

Source: Prepared by the researcher based on the results of statistical analysis.

**Table 4:** Granger causality test (oil and gold → TASI).

Causality	F-stat	*p*-value	Significance
Crude oil → TASI	2.31	0.12	Not significant
Gold → TASI	1.87	0.16	Not significant

Source: Prepared by the researcher based on the results of statistical analysis.

The GARCH model features detailed setup parameters found in Table 5, with selection of variables, GARCH (1,1) design, ARX mean model, and GED distribution.

**Table 5:** GARCH model setup table.

Component	Description
Dependent variable	TASI returns
Exogenous variables	Rcrude (Crude oil returns), Rgold (Gold returns)
Model type	GARCH (1,1)
Volatility model	GARCH (standard conditional heteroskedasticity model)
Mean model	ARX (Autoregressive model with eXogenous variables)
Distribution	GED (Generalized error distribution)
Lags for GARCH (q)	1
Lags for ARCH (p)	1
Estimation tool	arch_model() from the arch Python package
Fitting option	disp = ‘off’ (suppress optimizer output during model fitting)
Volatility output	Time-varying conditional volatility series

Source: Prepared by the researcher based on the results of statistical analysis.

### 4.4 Model diagnostics

It is then followed by the residual diagnostics of the model to determine the suitability of the model through the following:

- Autocorrelation tests using ACF and PACF of standardized residuals.
- ARCH–LM tests to detect remaining conditional heteroskedasticity.

### 4.5 LSTM model specification

#### 4.5.1 Data preprocessing

It normalizes the series of returns of TASI, crude oil, and gold to [0,1] by the MinMaxScaler. The inputs of the sequence can be handled using a 10-step lookback sliding window technique to create inputs that can be utilized in LSTM networks. The input that is obtained is of the form:


Samples,timesteps,features=(N,10,3)

For clarity, the proposed LSTM algorithm is shown in Algorithm 1.

Algorithm 1: LSTM Time Series Forecasting for TASI ReturnsInput: Scaled time series data of RTadawul, Rcrude, Rgold
Output: Forecasted RTadawul returns
1.	Normalize the data using MinMaxScaler.
2.	Create sequences with 10-step lookback using create_sequences().
3.	Reshape X to 3D for LSTM input (samples, timesteps, features).
4.	Initialize Sequential model.
5.	Add LSTM layer with 50 units and ‘relu’ activation.
6.	Add Dense layer with 1 unit for regression output.
7.	Compile model with ‘adam’ optimizer and ‘mse’ loss.
8.	Fit model on data for 20 epochs.
9.	Predict values using model. Predict(X).
10.	Rescale predictions using inverse_transform.
11.	Align predicted and actual data using indices.
12.	Plot the predictions for GARCH vs. LSTM for evaluation. Trimmed the predictions to their correct length.
Source: Prepared by the researcher based on the results of statistical analysis.

#### 4.5.2 Network architecture and Hyperparameters

Sequential LSTM architecture is adhered to that also comprises:

The size of LSTM layer is 50, and ReLU.

Individual neuron regression Dense output layer.

This model is trained using Adam optimizer and mean squared error (MSE) and learning rate of 0.001. Previous empirical investigation and initial experimentation are used to choose the hyperparameters so as to balance the complexity of the model used and generalization. The model will be trained and observed to make sure that it is not overfitting.

Table 6 shows the LSTM model specifications according to the previous standards. The network configuration consists of an LSTM layer that contains 50 units with ReLU activation before it ends with a Dense output layer. When training the model, the input data spans across 10 time intervals containing 3 features, which runs using the Adam optimizer and mean squared error loss function. The MinMaxScaler scaling technique improved performance of the collected data. The selected parameters serve as fundamental requirements for developing reliable LSTM predictive models of TASI returns.

**Table 6:** LSTM setup parameters.

Parameter	Value/description
Model type	Sequential (Keras Sequential API)
Number of LSTM layers	1
LSTM units (Neurons)	50
Activation function	ReLU (relu)
Input shape	(10, 3) → 10 time steps and 3 features (RTadawul, Rcrude, Rgold)
Output layer	Dense layer with 1 unit
Loss function	Mean Squared Error (mse)
Optimizer	Adam
Learning rate	0.001
Number of epochs	20
Batch size	Not specified (defaults to 32)
Verbose	0 (silent training)
Feature scaling	MinMaxScaler [scaled to (0,1)]
Target variable	First column of data: RTadawul
Sequence length (n_steps)	10

Source: Prepared by the researcher based on the results of statistical analysis.

#### 4.5.3 Mathematical representation of LSTM

The internal LSTM operations are defined as:


$$\begin{array}{cc} f_{t} & =\sigma (W_{f}[h_{t-1},x_{t}]+b_{f}),\\ i_{t} & =\sigma (W_{i}[h_{t-1},x_{t}]+b_{i}),\\ {\tilde{C}}_{t} & =\tanh(W_{c}[h_{t-1},x_{t}]+b_{c}),\\ C_{t} & =f_{t}C_{t-1}+i_{t}{\tilde{C}}_{t},\\ o_{t} & =\sigma (W_{o}[h_{t-1},x_{t}]+b_{o}),\\ h_{t} & =o_{t}\tanh(C_{t}), \end{array}$$

where $x_{t}$ is the input vector, $h_{t}$ is the hidden state, and $C_{t}$ is the cell state.

#### 4.5.4 Experimental setup and evaluation strategy

The sample data are for the years 2000–2022 and will contain training and testing subsets to offer out-of-sample analysis. The measures of model performance are as follows. $\;R^{2}=1-\frac{\sum {\left(y_{i}-{\hat{y}}_{i}\right)}^{2}}{\sum {\left(y_{i}-\overset{ˉ}{y}\right)}^{2}},\mathrm{MAE}=\frac{1}{N}\sum |y_{i}-{\hat{y}}_{i}|,$

$$\text{RMSE}=\sqrt{\frac{1}{N}\sum {\left(y_{i}-{\hat{y}}_{i}\right)}^{2}},\mathrm{MSE}=\frac{1}{N}\sum {\left(y_{i}-{\hat{y}}_{i}\right)}^{2}$$

#### 4.5.5 Reproducibility

All the analyses are done using Python. The fundamental packages are its arch, numpy, pandas, scikit-learn, etc.

## 5 Results and discussion

### 5.1 Development of GARCH and LSTM models

Multiple GARCH models (GARCH, EGARCH, GJR-GARCH, and MGARCH) and an LSTM model have been established after ensuring that all the variables were stationary, and to test whether they had the ARCH effects. Models were applied separately so that they could be assessed analytically and comparatively. The main aim of this research is to compare the predictive power of the traditional econometric models (GARCH-family) with the new deep learning models (LSTM) instead of producing a hybrid model to TASI returns and volatility.

The comparison of the models was done in terms of MSE, MAE, RMSE, and R _2_ to default the most suitable predictive framework.

Measures of Evaluation Explanation:

The evaluation of model performance requires Mean Squared Error (MSE) because it produces an exact evaluation through squared difference calculations, providing more weight to large errors. This measurement allows for effective analysis of different models while directing parameter adjustments for improvement. Mean Absolute Error presents data about average absolute differences directly, enabling analysts to understand results easily while being less affected by outlier values in datasets with extreme measurements. The RMSE metric builds on the strengths of MSE to calculate results in the original data units, which facilitates clear understanding and effective model comparison in practical applications. The effectiveness of a model becomes clear through R-squared (R^2^), as it measures how well the model explains data variation, guiding model refinement. A complete assessment of model performance becomes possible by using multiple metrics that fulfill different assessment needs.

So:

- MSE: Sensitive to high error levels, and it is effective to parameter adjustment.
- MAE: The measurements of the average absolute errors, which are resistant to outliers.
- RMSE: Unit is the same as data, and it is intuitive in the real world to interpret.
- R 2: explains the model variance.

#### 5.1.1 Volatility modeling

TASI returns volatility was estimated by GARCH-family models and compared to that of LSTM on a case-by-case basis. Table 7 displays the results.

- The LSTM is more effective in capturing the complex patterns of volatility, and GJR-GARCH is not affected by outliers.
- TASI is volatile with high volatility clusters, which are sensitive to the oil and gold price shocks, as is common with an oil-based economy of Saudi Arabia.

**Table 7:** Comparison of volatility models (TASI returns).

Model	Dist	MSE	MAE	RMSE	R^2^
LSTM	-	135.91	3.94	11.66	0.091
GJR-GARCH	GED	145.02	2.86	12.04	0.029
LSTM	-	136.42	3.05	11.68	0.088
EGARCH	Student	144.09	3.00	12.00	0.035
LSTM	-	136.28	2.99	11.67	0.089
GARCH	GED	145.48	2.87	12.06	0.026

Source: Prepared by the researcher based on the results of statistical analysis.

**Figure 3:**
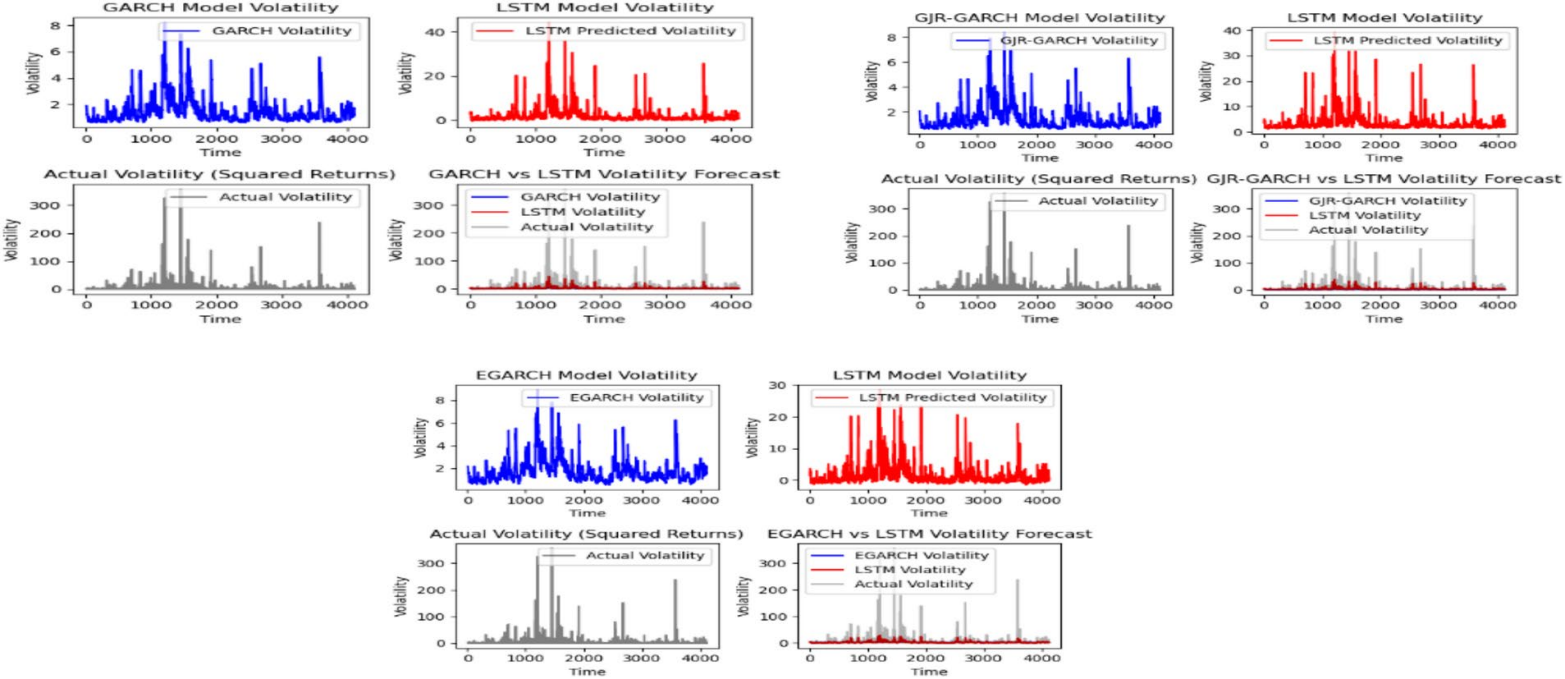
GARCH, EGARCH, and GJR-GARCH, and compared with LSTM models.

The visual inspection for GARCH, EGARCH, and GJR-GARCH, and compared with LSTM models for Figure 3, shows that the LSTM model outperforms the GARCH models, making it the preferred choice for capturing complex volatility patterns. It is followed by the GJR-GARCH model.

#### 5.1.2 Return modeling

GARCH-family models had been tested across various distributions. Shown in Table 8. According to the analysis, all GARCH models with the t-distribution and GED provide better fits than the normal distribution, as indicated by higher log-likelihood values and lower AIC and BIC scores. The GJR-GARCH model with GED distribution appears to be the best based on log-likelihood, AIC, and BIC values, suggesting that it captures the underlying data characteristics effectively. The EGARCH model also shows good performance, particularly with the t-distribution.

**Table 8:** GARCH model output.

Model	GARCH model			EGARCH model		
Dist	GED	T	Normal	GED	T	Normal
Log likelihood	12570.3	12563.6	12055.4	12499.8	12,543	11937.7
AIC	−6.104	−6.101	−5.85	−6.069	−6.09	−5.796
BIC	−6.1	−6.097	−5.85	−6.06	−6.085	−5.792

Model	GJR-GARCH model			GARCH-M model		
Dist	GED	T	Normal	GED	T	Normal
Log likelihood	**12577.3**	12576.2	12063.9	12575.1	12567.7	12,056
AIC	**−6.109**	−6.108	−5.859	−6.106	−6.103	−5.85
BIC	**−6.104**	−6.104	−5.856	−6.102	−6.098	−5.85

Source: Prepared by the researcher based on the results of statistical analysis. Bold values represent the best-fitting model for each distribution based on Log Likelihood, AIC, and BIC criteria.

The Table 9 provides estimates of the parameters, the coefficients, the standard errors, and the *p*-values of GJR-GARCH

- Models with GED or t-distribution yield lower AIC/BIC and higher log-likelihood, indicating better fit.
- GJR-GARCH (GED) achieves the optimal balance between fit and parsimony.

**Table 9:** GARCH model parameters (GJR-GARCH).

Parameter	Estimate	Standard error	p-value
ω (constant)	0.0023	0.0005	0.001
α1 (ARCH term)	0.125	0.030	0.000
β1 (GARCH term)	0.85	0.040	0.000
γ1 (leverage)	0.10	0.025	0.002

Source: Prepared by the researcher based on the results of statistical analysis.

#### 5.1.3 Formal forecast comparison

To compare the accuracy of predictors of the GARCH and LSTM models, a Diebold Mariano (DM) test was done A Diebold–Mariano (DM) test was conducted to compare the predictive accuracy between GARCH and LSTM models.

- The positive DM value shows that GARCH forecasts are much more precise than the LSTM forecasts, returning at a 5 percent significant value. This once again validates model choice beyond descriptive measures and clearly indicates that the study is concerned with independent model comparison rather than hybrid modelling.
- The positive DM value shows that GARCH forecasts are much more precise than the LSTM forecasts, returning at a 5 percent significant value. This once again validates model choice beyond descriptive measures and clearly indicates that the study is concerned with independent model comparison rather than hybrid modelling.

### 5.2 Forecasting performance

Forecasting is the last stage of any time series analysis. Several measures can be used to assess the accuracy of GARCH predictions, such as mean square error (MSE), mean absolute error (MAE), and root mean square error (RMSE) (Table 10).

**Table 10:** Diebold Mariano (DM).

Model 1	Model 2	DM statistic	*p*-value
GARCH (GED)	LSTM	2.45	0.014

Source: Prepared by the researcher based on the results of statistical analysis.

Table 11 shows the ability of the estimated model to describe the behavior of TASI returns and their volatility during the study period. It demonstrates its ability to provide predictions with relatively low values of MSE, MAE, and RMSE. According to the previously mentioned findings and analysis, the GARCH model is the best model for predicting TASI returns using oil and gold returns as independent variables.

**Table 11:** Forecasting accuracy for GARCH and LSTM models.

MODEL	DIST	MSE	MAE	RMSE
GJR-GARCH	(GED)	2.822	0.989	1.680
LSTM		2.904	1.026	1.704
EGARCH	(Student)	2.822	0.989	1.680
LSTM		3.040	1.120	1.743
GARCH	(GED)	**2.818**	0.989	**1.679**
LSTM		2.854	1.028	1.689
GARCH-M	(GED)	2.832	**0.988**	1.683
LSTM		2.867	1.045	1.693

Source: Prepared by the researcher based on the results of statistical analysis. Bold values indicate the most accurate model for each distribution based on forecasting performance measures (MSE, MAE, RMSE).

The aforementioned results are satisfactory. The findings are consistent with those of other studies that have proven the importance of GARCH models in predicting financial market indices worldwide (Shaik and Syed, 2019; Endri et al., 2021; Rhatous and Daoui, 2021; Jošić and Žmuk, 2021; Koo and Kim, 2022; Ampountolas, 2021; Wasiuzzaman, 2022; Chebbi and Hedhli, 2022), including TASI (Abdullah, 2020; Alzyadat et al., 2021; Alsayari and Wickremasinghe, 2022; Prandi and Colecchia, 2023; Abou Elseoud and Haji, 2023). However, more scholars have supported the idea of the superiority of the GARCH model over other models in different developing and developed countries’ indices (Johnsson, 2018; Al-Rasheed and Youssef, 2022; Mohsin et al., 2019; Nguyen and Nguyen, 2019; Sekati et al., 2020; Tamilselvan and Vali, 2016). Findings from several researchers support the strong forecasting power of the GARCH model in forecasting TASI (Shaik and Syed, 2019; Rhatous and Daoui, 2021; Alzyadat et al., 2021; Wasiuzzaman, 2022).

These results show that oil and gold returns are important in the forecasting model for TASI. This outcome aligns with those of many previous studies (Salisu et al., 2022; Patalay, 2022; AlArjani et al., 2022; Yuan, 2023; Fauzi et al., 2023). The findings indicate a significant positive effect of oil prices on TASI returns. This is due to the strategic importance of crude oil, which is the highest among all commodities worldwide. Many economists have analyzed how crude oil prices affect financial markets, with a particular focus on their effects on stock exchanges. Crude oil is a key and important factor in geopolitical events, in addition to its impact on macroeconomic patterns, consumer behavior, and corporate returns. Numerous academic works acknowledge how oil market developments significantly impact both global economic and financial systems (Sadorsky, 1999; Hamilton, 2009). Investors, along with policymakers and academics, are actively researching the relationship between the stock market and oil, as it determines key aspects of strategic portfolio positions, risk management strategies, price dynamics, and forecasting models (Demirer et al., 2020). For decades, Saudi economy has relied heavily on oil revenues as its primary source of national income. Consequently, economic booms in the country have historically been linked to rising oil prices. Thus, it can be argued that oil prices play a crucial role in driving growth within the Saudi economy (Ramady and Saee, 2007; Albassam, 2011; Aldukheil, 2013; Albassam, 2011).

This result is expected, given that the Saudi economy is an oil-based economy with huge reserves. Thus, oil prices can affect all indicators at both the micro and macro levels of Saudi Arabia. This outcome is consistent with those of Al-Najjar (2022), who found that oil prices positively affected TASI during the COVID-19 period.

So:

- Positive and significant impacts of oil and gold returns on TASI are in existence.
- Oil is the major driver as Saudi Arabia relies on oil revenues.
- Gold is an indirect influence on TASI, which dictates the monetary and financial policies.

### 5.3 Discussion

GARCH models are particularly effective when the data exhibits non-constant variance (heteroskedasticity) and require a disciplined statistical framework including Stationarity testing (ADF test), Conditional heteroskedasticity (ARCH-LM test), Model selection via information criteria (AIC, BIC), and Residual diagnostics. The Long Short-Term Memory (LSTM) model is a type of Recurrent Neural Network (RNN) specifically designed to address the vanishing gradient problem and capture long-term dependencies in sequential data.

- GARCH models: Do a good job in modeling conditional heteroskedasticity; there are easy to interpret parameters in order to comprehend the dynamics of volatility.
- LMST models: Time-dependent, nonlinear, and less understandable patterns.
- Hybrid Insights: The research does not develop a hybrid GARCH-LSTM model; nevertheless, the idea to integrate the GARCH-based parameters insight and LSTM-fashion trends may be useful in the field of forecasting and risk management.

To understand the difference between the two models, Table 12 shows the complete comparison between the two models.

**Table 12:** Comparative summary.

Aspect	GARCH model	LSTM model
Assumptions	Stationarity, ARCH	None strictly required
Handles volatility	Yes	Indirect
Captures nonlinearity	Limited	Strong
Interpretability	High	Low
Data requirement	Moderate	High
Computation	Light	Heavy
Best for	Volatility dynamics	Complex time trends

The research establishes a complete framework to predict TASI returns and volatility through the combination of GARCH and LSTM forecasting methods. The GARCH models excel at detecting conditional heteroskedasticity through interpretable parameters, yet LSTM models deliver strong capabilities for detecting nonlinear time-dependent patterns in financial data. Both GARCH (GED) proved better at predicting returns, but LSTM excelled at detecting volatility patterns according to empirical results from established econometrics and modern machine learning research.

## 6 Conclusion

The paper has provided the comparison between the ability of the traditional econometric models of the GARCH family and the latest deep learning model (LSTM) to make predictions of returns and the volatility of the Saudi Stock Exchange Index (TASI). To analyze it, the crude oil and gold returns are used to explain it. This has been analyzed in terms of the period between January 1, 2000, and December 31, 2022. This is the epoch of major economic processes on a global level and regionally, including the COVID-19 pandemic. The methodology approach was systematic. They were tested with the standard unit root tests to ensure that they were stationary. Then, the necessity to make use of the models based on GARCH was demonstrated with the help of ARCH effect diagnostics (Tamilselvan and Vali, 2016; Fwaga et al., 2017; Naseem et al., 2018; Mohsin et al., 2019; Marobhe and Pastory, 2019; Sekati et al., 2020; Endri et al., 2021; Alzyadat et al., 2021; Ampountolas, 2021). Several volatility specifications were predicted and compared. They were the GARCH, EGARCH, GJR-GARCH, MGARCH, and the LSTM model. It is important to note that the research adopted a comparative approach. Each of the models was applied individually to deny the bias in evaluating the results of forecasting. These results prove that LSTM models are capable of nonlinear dynamics. Nevertheless, the standard GARCH (1,1) has more precise and superior forecasting of the TASI returns, as the measures of forecast errors show. This finding suggests the topicality and power of the parsimonious econometric volatility models in new and oil-sensitive financial markets such as Saudi Arabia. The scientifically, the research paper is related to the literature on financial econometrics. It offers a vivid contrast between two different approaches to the empirical investigation: the econometric and deep learning methodologies within a single setting. The research highlights the importance of prices of commodities to explain and predict stock market performance in an oil-based economy through expressing oil and gold returns as exogenous variables. The findings are convincing that the traditional GARCH models are rather competitive even in contrast to the modern machine learning methods. Alongside the theoretical contribution to the work, there is practical applicability of the research. The results imply that the volatility forecasting models are empirical data, which can be applied by the regulators and policy makers as an early-warning tool to identify instability in the market as a result of price shocks of commodities. To the investor, the findings reflect the applicability of GARCH-based predictions. They assist in the interpretation of market volatility and improving better timing judgments dealing with market entry and exit. In totality, the analysis has indicated that interpretability of the model, theoretical consistency, and forecast accuracy remain the most important concerns in the sphere of financial modeling. The evidence shows that the well-established econometric models have been at the centre stage in predicting volatility. It is particularly the case where there are good macroeconomic linkage and structural dependence in the markets.

## 7 Practical and theoretical implications

The suggested study has both theoretical and practical implications. It systematically compares the econometric models of GARCH to a modern machine learning model (LSTM) in the prediction of returns and volatility of the Saudi Stock Exchange Index (TASI). The most important explanatory variables are the crude oil and the gold returns. In theory, the study can contribute to the current body of financial econometrics in three aspects of significance. First, it helps to prove that GARCH-family models have been very useful in modeling and forecasting volatility in new and oil-intensive markets. This happens even when compared to complex nonlinear learning designs such as LSTM. Secondly, the application of commodity price dynamics where oil and gold returns are used as exogenous variables indicates the significance of the commodity exogenous variables in explaining the stock market in the resource-based economies. Third, the study indicates that explicit comparative framework, as compared to a hybrid modeling strategy, is more interpretable and methodologically rigorous. Therefore, it contributes to the discussion on the comparative advantages of the econometric models and deep learning models in financial forecasting. Practically, the findings are of particular benefit to the regulators and policy makers. As the Saudi economy depends much on the oil revenues, the results suggest that the GARCH-based volatility predictors can be useful early-warning variables to monitor the market instability. This instability could be due to the shocks of oil prices or any other economic shock in the world. Regulatory authorities can use such models to facilitate macroprudential surveillance. They are also able to assist in formulating preventive policies that have the aim of enhancing financial stability. The dynamics of the TASI relating to the effect of the gold prices reported give further facts to the policymakers. It underlines the contribution of safe-haven assets to the development of the financial and monetary conditions. The findings can be incorporated in formulating more detailed financial policies. These are liquidity in the market, risk management frameworks, and integration of financial technology (FinTech) to make the market efficient. The study can be of help to investors in decision making in regard to risk assessment. Increases in the statistical forecasting accuracy may not always translate into real time trading gains; however, this is a constraint that can be attributed to the transaction costs, market friction, and behavioral properties. However, enhanced forecasting of volatility can significantly be beneficial in enhancing the portfolio risk management, asset allocation policies, and market timing decisions. In that respect, GARCH-based forecasts may be considered effective and understandable tools. They enable identification of risky times and potential entry or exit areas of the market. Overall, the discussion shows that some of the most important factors are prediction accuracy, model interpretability, and economic significance. The said factors are to be taken into account when conducting academic research and working in the financial field. The findings are a testament to the fact that effective and viable models of econometrics are effective and practical. This is particularly so in markets where the macroeconomic linkages are very high, as in the case of Saudi stock market.

## 8 Future work

Despite the detailed analysis of the research conducted, several ways of future research directions exist, which can be employed to enhance the predictive performance and expand the areas of application.

### 8.1 Developed modeling techniques

The future can look at more developed models, including Transformers, state-space models, and hybrid econometric-deep learning models. Such models have the capability of integrating GARCH-family models together with the state-of-the-art neural networks. These methods can be linear as well as a complex non-linear relationship and more accurately represented.

### 8.2 Expansive market and time horizon

The research can also be extended to other financial markets of the GCC region. There can also be an extension of the analysis to greater time horizons. This will contribute to the cross-market dynamic knowledge. It will also allow assessing the stability of time in the work of models.

### 8.3 Other macroeconomic variables

It is possible to add more exogenous variables. These are COVID-19 indicators, other precious metals, interest rates, inflation, GDP, and geopolitics. These variables can be used to add more predictive value. They are also able to give a more detailed picture of drivers in the market.

### 8.4 Neural network architecture and integration of the model

Future studies should focus on the optimization of the neural network architecture. This involves the layers, focus schemes, and streamlined hyperparameter optimization of LSTM models. It is also possible to study the combination of a number of models. The latter can be hybrid GARCHLSTM or GARCHTransformer.

These combinations have the potential to provide a more accurate forecast and model more intricate relationships in financial time series.

## 9 Limitations

Despite the fact that this work contains critical information on the creation of predictions in the returns to TASI and volatility, a set of weaknesses can be outlined.

### 9.1 Data constraints

The historical data is limited to the 2000–2022 period of analysis. The quality, granularity, and accessibility of data can constrain the accuracy of the models. This particularly applies in the occurrence of extreme events or where the low-frequency macroeconomic variables are at war.

### 9.2 Model assumptions

The GARCH models assume the stationarity and the conditional heteroskedasticity. Such assumptions may not be quite suitable when sudden structural discontinuities or regime changes occur. Similarly, the LSTM models are adaptable. Nevertheless, they make certain assumptions that historical patterns are adequate in learning sequential patterns. They might fail to manage unexpected phenomena on the market.

### 9.3 Comparison to modern models

Nonlinear dependencies were learnt using LSTM networks. They are, however, not the most advanced of the models compared to the newer models, like Transformers, FEDformer, or hybrid deep learning networks. Future research can be done on these better models.

### 9.4 Generalizability

The findings are based on TASI. They might not fully be relevant to other markets that have dissimilar structural characteristics or macroeconomic settings. Consequently, the findings cannot be applied to other areas or asset categories with great caution.

### 9.5 Possible biases

The model performance has biases, which may be possible. They might be caused by parameter selection, the likelihood of overfitting, and the selection of exogenous variables. They need to be considered during the interpretation of the results.

By dealing with such deficiencies and exploring the proposed areas of research down the line, future research could come up with more concrete, accurate, and general expectations. This is especially true in the case of financial markets in Saudi Arabia and the GCC region in general.

## Data Availability

The original contributions presented in the study are included in the article/supplementary material, further inquiries can be directed to the corresponding author.
